# Protective effects of a triple‐fermented barley extract (FBe) against HCl/EtOH‐induced gastric mucosa damage in mice

**DOI:** 10.1002/fsn3.745

**Published:** 2018-10-01

**Authors:** Jong‐Min Lim, Chang‐Hyun Song, Su‐Jin Park, Dong‐Chan Park, Hyung‐Rae Cho, Go‐Woon Jung, Khawaja Muhammad Imran Bashir, Sae Kwang Ku, Jae‐Suk Choi

**Affiliations:** ^1^ #305 Marine Bio‐Industry Development Center Glucan Corp. Gijan‐gun Busan Korea; ^2^ Department of Anatomy and Histology College of Korean Medicine Daegu Haany University Gyeongsan‐si Gyeongsanbuk‐do Korea; ^3^ MRC‐GHF, College of Korean Medicine Daegu Haany University Gyeongsan‐si Gyeongsanbuk‐do Korea; ^4^ Seafood Research Center, IACF Silla University Seo‐gu Busan Korea; ^5^ Research Center for Extremophiles and Microbiology College of Medical and Life Sciences Silla University Sasang‐gu Busan Korea; ^6^ Division of Bioindustry College of Medical and Life Sciences Silla University Sasang‐gu Busan Korea

**Keywords:** gastroprotective effects, HCl/EtOH‐induced gastric ulcer, mice, triple‐fermented barley extracts (FBe)

## Abstract

This study was designed to observe the possible protective effects of a triple‐fermented barley (*Hordeum vulgare* L.) extract (FBe) obtained by saccharification and using *Saccharomyces cerevisiae* and *Weissella cibaria* in alleviating gastric damage induced by a hydrochloric acid (HCl) and ethanol (EtOH) mixture in mice. After oral administration of FBe (300, 200, and 100 mg/kg) followed by 1 hr before and after the single treatment of HCl/EtOH (H/E) mixture, the hemorrhagic lesion scores, histopathology of the stomach, gastric nitrate/nitrite content, lipid peroxidation, and antioxidant defense systems including catalase and superoxide dismutase activities were observed. Following a single oral treatment of H/E‐induced gastric damages as measured by hemorrhagic gross lesions and histopathological gastric, ulcerative lesions were significantly and dose‐dependently (*p* < 0.01 or *p* < 0.05) inhibited in mice, when all three different doses of FBe were administered as compared to those in H/E control mice. In particular, FBe also increased gastric nitrate/nitrite content and strengthened the antioxidant defense, with a decrease in the level of gastric lipid peroxidation, but increased the activities of CAT and SOD. Moreover, the effects of FBe are comparable to that of ranitidine, a reference drug. The obtained results suggest that this fermented barley extract prevented mice from H/E‐induced gastric mucosal damages through the suppression of inflammatory responses and oxidative stress‐responsive free radicals. Thus, FBe can be useful to treat patients suffering from gastric mucosal disorders as a potent food supplement, and thereby, it would increase the necessity of application in the food industry.

## 1 INTRODUCTION

Gastric ulcers affect a number of people worldwide (Oyagi, Ogawa, Kakino, & Hara, 2010). The prevalence of this disease can lead to occur more often due to excessive drinking of alcohol, smoking, stress, infections, nutritional deficiency, abrupt use of nonsteroidal drugs for managing anti‐inflammatory diseases (NSAIDs); it is a multietiologic chronic disease (Alqasoumi, Al‐Sohaibani, Al‐Howiriny, Al‐Yahya, & Rafatullah, 2009; Gisbert, 2005; Süleyman et al., 2009). Pathologically, digestive ulcers located in the gastric and duodenal mucosa induce acute erosive and surface desquamatious ulcerative lesions following exposure to gastric juices, including gastric acid and pepsin (Alqasoumi et al., 2009; Huh et al., 2003). Disorders or decreases in gastric mucosa antioxidant defense systems have been implicated in the pathogenesis and progression of various gastric ulcers (Kwiecień, Brzozowski, Konturek, & Konturek, 2002). Apparently, the free radical scavenging property of drugs may contribute to reduce the progression of such oxidative damage to gastric mucosa (Bhattacharya, Chaudhuri, Chattopadhyay, & Bandyopadhyay, 2007; Oyagi et al., 2010). It has been accepted that free radical scavengers exert protective functions on mucosal damage triggered by excessive production of free radicals (Ahn et al., 2001; Cantürk, Cantürk, Ozbilim, & Yenisey, 2000; La Casa, Villegas, Alarcón de la Lastra, Motilva, & Martín Calero, 2000; Yoshinaga, Ohtani, Harada, Fukuda, & Nawata, 2001). Therefore, potent antiulcer agents derived from natural herbs, such as *Artemisia asiatica* (DA‐9601; Stillen^™^, Dong‐A Pharmaceuticals, Yongin, Korea), are being sold in markets based on their potent antioxidant effects (Baek et al., 2011).

Use of EtOH in in vivo study for inducing gastric ulcer has been commonly used model systems to study on ulcerative pathogenesis and its curative measures (Szabo & Brown, 1987). In addition, to evoke severe and rapid gastric damage induced by EtOH, hydrochloric acid (HCl), which also involved in the progression and pathogenesis of gastric damages, has been additionally administered (Alqasoumi et al., 2009; Huh et al., 2003; Lee et al., 2010; Oyagi et al., 2010). Therefore, administration of the HCl/EtOH mixture has been used as a valuable and simple method to study both the pathogenesis of and the therapy for human ulcerative disease in mouse gastric ulcer models, especially to study natural products based on their potent antioxidant effects (Murata et al., 2012; Niero et al., 2012; Oyagi et al., 2010; Yang et al., 2012).

Some synthetic antiulcer drugs are reported to be used for commercial purposes such as misopostol and Ranitidine (RA), etc. However, misopostol has provided various adverse effects such as metabolism inhibitory effects of proton pump inhibitors (PPIs), headaches, antiandrogenic effects of H_2_ receptor blockers, dizziness associated with sucralfate, stillbirth, and melena in pregnant women (Miederer, 1986). Whereas, RA is a histamine H_2_ receptor antagonist affecting the inhibited stomach acid production (Grover, Adiga, Vats, & Rathi, 2001) and currently regarded as favorable free radical scavengers (Ahmadi et al., 2011). Although, it is likely to decrease mucosal perfusion in patients with acute renal or cardiac failure, and thereby increases the risk of death (Jakob, Parviainen, Ruokonen, Uusaro, & Takala, 2005). Infectious diarrhea (Neal, Briji, Slack, Hawkey, & Logan, 1994; Ruddell, Axon, Findlay, Bartholomew, & Hill, 1980), developing food allergies (Untersmayr et al., 2005), and thrombocytopenia (Bangia, Kamath, & Mohan, 2011) have also been reported as common side effects of histamine H_2_ receptor antagonists (Yesilada & Gurbuz, 2003). In this experiment, RA 100 mg/kg was selected as a positive reference drug to compare the gastric mucosa protective effects based on the previous report (Grover et al., 2001). These side effects are leading us to pay special attention to develop a nontoxic and inexpensive antiulcer medication (Yesilada & Gurbuz, 2003).

Globally, barley is considered a nontoxic plant (Robles‐Escajeda et al., 2013) that produces a cereal grain that serves as a base malt in the brewing industry. It is also a healthy component of various foods and beverages and a major animal feed (Robles‐Escajeda et al., 2013). Barley (*Hordeum vulgare* L), particularly, contains various phenolic compounds, which have been shown to have antioxidant activity and to be effective for improving health (Giriwono, Shirakawa, Hokazono, Goto, & Komai, 2011; Mattila, Pihlava, & Hellström, 2005; Pérez‐Jiménez & Saura‐Calixto, 2005), including anticancer (Robles‐Escajeda et al., 2013) and probiotic gastroprotective (Charalampopoulos, Pandiella, & Webb, 2003) activities. Fermentation procedures can lead to increase the availability of secondary metabolites and functionality of these phenolic compounds (Ye, Morimura, Han, Shigematsu, & Kida, 2004; Yoshimoto et al., 2004), especially the antioxidative effects (Giriwono et al., 2010, 2011). A number of barley extracts produced from fermentation have been reported to be potent pharmacological effects, especially antioxidant (Giriwono et al., 2011), uric acid lowering (Hokazono, Omori, Yamamoto, Akaoka, & Ono, 2010), antiatopic dermatitis (Hokazono, Omori, & Ono, 2010; Iguchi, Kawata, Watanabe, Mazumder, & Tanabe, 2009), hepatoprotective (Giriwono et al., 2010, 2011), and immunostimulatory (Kim et al., 2007) activities, compared with nonfermented extracts. In our previous reports on the laxative effects of fermented barley extract (FBe) in normal rats (Lim et al., 2018a) and loperamide‐induced constipation rats (Lim et al., 2018b), in which FBe showed favorable laxative effects at concentrations of 300, 200, and 100 mg/kg. However, there are no systemic assessments for gastroprotective effects of fermented barley extracts, to the best of our knowledge.

## 2 MATERIALS AND METHODS

### 2.1 Animals and husbandry

**Figure 1:**
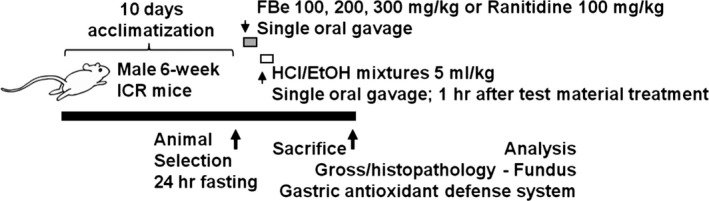
Experimental design used in this study. FBe: Triple‐fermented barley extracts, test material; HCl: Hydrochloric acid; EtOH: Ethanol; H/E: HCl/EtOH (98% EtOH contains 150 mM HCl) mixture; RA: Ranitidine hydrochloride

The mice (ICR; *N* = 60), healthy male, mean 38.27 ± 1.81 g, range 25.60–41.80 g, were purchased from OrientBio company (Seungnam, Korea) and used after 10 days of acclimatization. The animals were housed in a polycarbonate cage with maintaining the temperature 20–25°C, humidity 30%–35%, 12‐hr light:dark cycle, and ad libitum of water and food. Eight mice for each of the six groups, 48 mice in total, were selected based on body weight (mean 38.27 ± 1.81 g, range 25.60–41.80 g) measured at 1 day before the test material administration after 10 days of acclimatization and were used for further experiments (Table 1 and Figure 1).

**Table 1:** Gastric lipid peroxidation, nitrate/nitrite contents, and catalase and SOD activities in the intact or HE‐treated mice

Items (unit) groups	Lipid peroxidation (nM of MDA/g tissue)	Nitrate/nitrite contents (μM/g protein)	Enzyme activity	
			Catalase (mM min^−1^ g tissue^−1^)	SOD (mM min^−1^ mg tissue^−1^)
Control				
Intact	2.16 ± 0.70	2.74 ± 0.75	624.25 ± 123.89	731.50 ± 146.04
HE	6.70 ± 0.90^a^	1.06 ± 0.25^e^	265.63 ± 53.87^a^	326.75 ± 67.09^a^
Reference				
RA 100 mg/kg	3.68 ± 0.68^ac^	1.79 ± 0.26^fg^	391.63 ± 50.81^ac^	465.75 ± 50.70^ac^
FBe treated as				
300 mg/kg	2.89 ± 0.68^c^	3.32 ± 0.61^g^	638.38 ± 117.29^c^	624.75 ± 111.80^bc^
200 mg/kg	3.81 ± 0.87^ac^	1.77 ± 0.24^eg^	386.25 ± 71.39^ac^	465.38 ± 55.73^ac^
100 mg/kg	4.92 ± 0.63^ac^	1.47 ± 0.28^e^ ^h^	350.00 ± 45.28^ad^	428.25 ± 43.11^ad^

Notes

Values are expressed mean ± *SD* of eight mice.

FBe: Triple‐fermented barley extracts, test material; HCl: Hydrochloride; EtOH: Ethanol; HE: HCl/EtOH (98% EtOH contains 150 mM HCl) mixture; RA: Ranitidine hydrochloride; MDA: Malondialdehyde; SOD: Superoxide dismutase.

^a^ *p* < 0.01 and ^b^ *p* < 0.05 as compared with intact control by LSD test.

^c^ *p* < 0.01 and ^d^ *p* < 0.05 as compared with HE control by LSD test.

^e^ *p* < 0.01 and ^f^ *p* < 0.05 as compared with intact control by MW test.

^g^ *p* < 0.01 and ^h^ *p* < 0.05 as compared with HE control by MW test.

Experimental groups (Six groups; eight mice in each group were finally sacrificed)

1. Intact control: Vehicle (distilled water 10 ml/kg)‐administered mice
2. H/E control: Vehicle and HCl/EtOH mixture‐treated, gastric ulcer‐induced vehicle control mice
3. RA: RA 100 mg/kg and HCl/EtOH mixture‐administered, reference drug‐treated mice
4. FBe 300: FBe 300 mg/kg and HCl/EtOH mixture‐treated, the highest dose experimental mice
5. FBe 200: FBe 200 mg/kg and HCl/EtOH mixture‐treated, the intermediate dose experimental mice
6. FBe 100: FBe 100 mg/kg and HCl/EtOH mixture‐treated, the lowest dose experimental mice

### 2.2 Preparation and administration of test articles

The FBe was prepared and supplied by Glucan Corp. (Busan, Korea) according to methods used in our previous studies (Choi, Kim, Cho, Kim, Lee, Ku, et al., 2014; Choi, Kim, Cho, Kim, Lee, Sohn, et al., 2014; Choi, Kim, Kim, Ku, et al., 2014; Choi, Kim, Kim, Lee, Sohn, et al., 2014; Lim et al., 2018a,b). Briefly, FBe was produced in three fermentation steps. In the first fermentation step, saccharification was performed by soaking nonglutinous rice (1 kg) for 6 hr, steaming for 15 min at 121°C and then fermenting at 55°C for 12 hr. In the second step, *Saccharomyces cerevisiae* (ATCC 9804, Manassas, VA, USA) was mixed to the first step resultant and incubated at 30°C for 48 hr. Finally, in the third fermentation step, lactic acid bacteria, *Weissella cibaria* (KACC 11845, Suwon, Korea), was mixed well to the second step resultant and incubated at 30°C for 48 hr. Specimens of FBe (Code FBe2014Ku01) were deposited in the herbarium of the Medical Research center for Globalization of Herbal Formulation, Daegu Haany University, Korea. FBe (100, 200, and 300 mg/kg) and RA were dissolved in distilled water, and orally administered at a volume of 10 ml/kg and 100 mg/kg, following reports of Lim et al. (2018a,b) and (Grover et al., 2001). The distilled water at 10 ml/kg was orally administered as negative control, as demonstrated in Table 1 and Figure 1. All chemicals were purchased from Sigma‐Aldrich, St. Louise, MO, USA, company unless otherwise stated. The RA was used as a potent reference drug, according to previous study (Grover et al., 2001).

### 2.3 Induction of gastric mucosa damage by the HCl/EtOH mixture in mice

One hour after treatment of the vehicle, three different doses of FBe, or RA 100 mg/kg to 24‐h fasted mice, an HCl/EtOH mixture (98% EtOH containing 150 mM HCl) was orally administered in a single volume of 5 ml/kg as reported previously (Oyagi et al., 2010). In intact vehicle control mice, sterilized distilled water was administered as a single gastric gavage, instead of the HCl/EtOH mixture. Both high‐grade HCl and EtOH were purchased from Merck (Darmstadt, Germany) and used after adjusting dilutions using distilled water (Table 1 and Figure 1).

### 2.4 Quantification of gross lesions

After sacrifice, the abdomen was opened with a median incision and the stomach was excised. The excised stomach was opened out along with greater curvature and fixed in 10% formalin solution for 24 hr before the acquisition of digital images. Ulcer areas on the stomach surface were examined microscopically and measured by an automated computer‐based image analyzer (iSolution FL ver 9.1, IMT i‐solution Inc., Quebec, Canada) according to the methods of Süleyman et al. (2009), Morais et al. (2010) and Oyagi Oyagi et al. (2010). For this purpose, the total area of the ulcerous stomach regions was calculated as mm^2^ of gastric mucosa.

### 2.5 Determination of lipid peroxidation or malondialdehyde (MDA) formation

The level of lipid peroxidation in the gastric mucosa was determined by estimating the MDA content following the thiobarbituric acid test (Ohkawa, Ohishi, & Yagi, 1979). The homogenate of mouse stomach was added to a solution containing sodium lauryl sulfate and acetic acid (Merck, Darmstadt, Germany), heated at 98°C for 1 hr, and then measured the supernatant at 532 nm wavelength using a UV/Vis spectrometer (OPTIZEN POP, Mecasys, Daejeon, Korea) which expressed as nM/g wet tissue.

### 2.6 Tissue CAT activity

CAT activity was determined by the method of Evans and Diplock (1991). A homogenate of mouse gastric mucosa was prepared, and the absorbance was measured at 240 nm for 100 s using a UV/Vis spectrometer. The results were expressed as mM min^−1^ mg tissue^−1^.

### 2.7 Tissue SOD activity

Gastric SOD activity was determined by a modified method of Minami and Yoshikawa (1979). Briefly, gastric homogenate was used to react with triscacodylate buffer (pH 8.2, 16% Triton X‐100, and nitroblue tetrazolium), incubated for 5 min at 37°C, and then measured at 540 nm using a spectrophotometer that expressed as mM min^−1^ mg wet tissue^−1^.

### 2.8 Gastric nitrate/nitrite contents

Gastric nitric oxide levels were measured as total nitrate/nitrite levels with the use of the Griess reagent (Green et al., 1982). Briefly, the stomach homogenate was mixed with Griess reagent and determined at wavelength of 540 nm using a microplate reader (Tecan, Männedorf, Switzerland). The resulting value was expressed as μM nitrate/nitrite per g of protein, where the protein concentration was detected using a Bradford assay method (Bradford, 1976).

### 2.9 Histopathology

Approximate regions of the stomach (between cardiac and pylorus, the fundus) were sampled and cross‐trimmed across the lumen. All trimmed fundus samples were fixed in 10% neutral buffered formalin (NBF) for 24 hr. After paraffin embedding, 3–4 μm sections were prepared. Representative sections were stained with hematoxylin and eosin (H&E) for examination by light microscopy. Subsequently, histological profiles of individual cross‐trimmed fundus tissues were observed. To further detail changes, the total thickness of the fundic mucosa, from the luminal mucosal surface to the muscularis mucosa on the periulcerative regions of the cross‐trimmed histological specimens, was measured using an automated computer‐based image analyzer under microscopy (Model Eclipse 80i, Nikon, Tokyo, Japan) as described by Ku et al. (2009). In addition, lesion invasive percentages in the fundus (%) were also calculated as follows in Equation (1) according to the method of Ku et al. (2009). Semiquantative scoring into four categories; 0 = normal intact mucosa, 1 = slight surface erosive damage, 2 = moderate mucosa damage, and 3 = severe total mucosa damage was based on general and histomorphometric analysis, aforementioned in this experiment.

(1) $$\begin{array}{cc} \text{Invasive Percentage of Lesions}\;(\%)\; & =\;(\text{Length of lesions on the cross}\\ & \;-\text{trimmed fundic walls}/\\ & \;\text{total thickness of}\\ & \;\text{cross‐trimmed fundic walls})\;\times \;100 \end{array}$$

### 2.10 Statistical analyses

All numerical data were expressed as mean ± SD of eight determinations. The obtained data were analyzed by one‐way ANOVA test followed by least‐significant differences (LSD) multicomparison test and Mann–Whitney U (MW) test (Ludbrook, 1997), using SPSS for Windows (Release 14.0K, SPSS Inc., Chicago, IL, USA). Differences were considered significant at *p* < 0.01 or *p* < 0.05. In addition, the percent‐point changes between intact and H/E control mice were calculated to observe the effects of severities of gastric mucosa damage including ulcerative lesions and the percent‐point changes as compared with H/E control and FBe or RA‐treated mice were also calculated to evaluate the effects of test substances as described by Equations (2) and (3), and our previous established method (Kang et al., 2014), respectively.

(2) $$\begin{array}{cc} & \text{Percentage Change Compared to the Intact Control}\;(\%)\;=\\ & \;(((\text{Data of H/E control}\\ & -\text{Data of intact control mice})/\text{Data of intact control mice})\;\times \;100) \end{array}$$

(3) $$\begin{array}{cc} & \text{Percentage Changes Compared to the H/E Control}\;(\%)\;=\\ & \;(((\text{Data of test substance treated mice}\\ & -\text{Data of H/E control mice})/\\ & \text{Data of H/E control mice})\;\times \;100) \end{array}$$

## 3 RESULTS

### 3.1 Changes in gastric mucosa gross lesions

**Figure 2:**
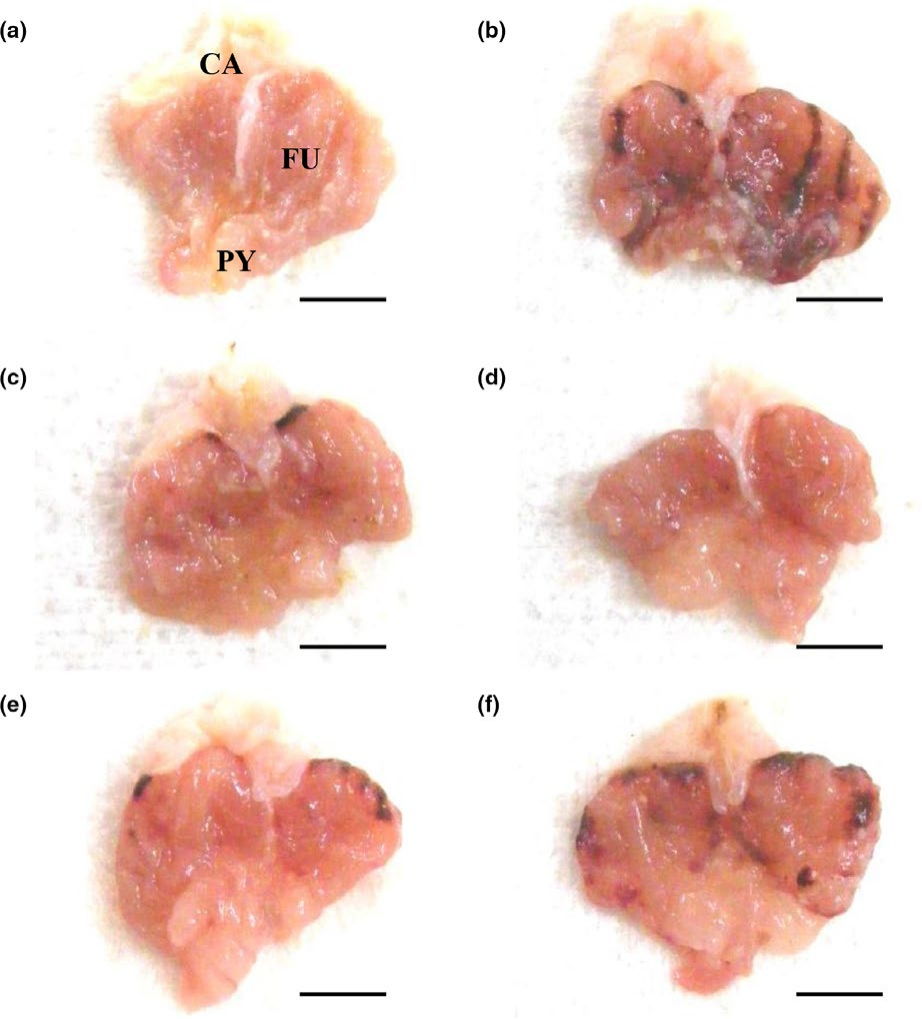
Representative gross stomach images, taken from intact or H/E‐treated mice. (a) Intact control (Distilled water 10 ml/kg and 5 ml/kg administrated mice). (b) H/E control (Distilled water 10 ml/kg and H/E 5 ml/kg‐treated vehicle control mice). (c) Reference (RA 100 mg/kg and H/E 5 ml/kg administrated reference drug‐treated mice). (d) FBe 300 (FBe 300 mg/kg and H/E 5 ml/kg treated the highest dosage experimental mice). (e) FBe 200 (FBe 200 mg/kg and H/E 5 ml/kg treated the highest dosage experimental mice). (f) FBe 100 (FBe 100 mg/kg and H/E 5 ml/kg treated the highest dosage experimental mice). FBe: Triple‐fermented barley extracts, test material; HCl: Hydrochloric acid; EtOH: Ethanol; H/E: HCl/EtOH (98% EtOH contains 150 mM HCl) mixture; RA: Ranitidine hydrochloride; CA: Cardiac regions of stomach; FU: Fundus regions of stomach; PY: Pylorus regions of stomach. All test substances were orally administered, at 1 hr before H/E 5 ml/kg single oral treatment, all animals were sacrificed at 1 hr after H/E treatment. Scale bars = 5 mm

**Figure 3:**
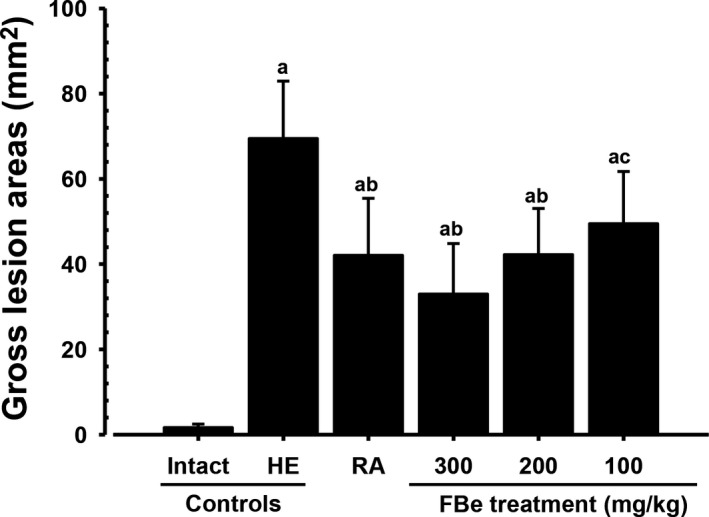
Gross lesion areas on the gastric mucosa, taken from intact or H/E‐treated mice. Values are expressed mean ± *SD* of eight mice, mm^2^. FBe: Triple‐fermented barley extracts, test material; RA: Ranitidine hydrochloride; HCl: Hydrochloric acid; EtOH: Ethanol; H/E: HCl/EtOH (98% EtOH contains 150 mM HCl) mixture. All test substances were single orally administered, at 1 hr before H/E 5 ml/kg single oral treatment, all animals were sacrificed at 1 hr after H/E treatment. ^a^ *p* < 0.01 as compared with intact control by MW test. ^b^ *p* < 0.01 and ^c^ *p* < 0.05 as compared with H/E control by MW test

Focal hemorrhagic ulcerative lesions were dispersed throughout the whole gastric mucosa in all HCl/EtOH mixture‐treated mice, but slight or negligible restricted ulcerative lesions were grossly observed in vehicle‐treated intact control mice. However, a noticeable and marked inhibition of the gross gastric damage was observed in RA (100 mg/kg)‐treated mice and in mice treated with all three doses of FBe (100, 200, and 300 mg/kg), dose‐dependently, compared to the observations in H/E control mice. Accordingly, significant (*p* < 0.01) increases in the gastric mucosa gross lesion area were detected in H/E control mice compared to that in intact control mice, but they were significantly (*p* < 0.01 or *p* < 0.05) decreased by treatment with any of the three doses of FBe and also by RA 100 mg/kg, dose‐dependently, compared to that in mice treated by H/E alone. Mice with FBe 200 mg/kg dosing had similar inhibitory activities on the increase in gastric mucosa gross lesion area induced by treatment with the HCl/EtOH mixture compared to RA 100 mg/kg (Figures 2 and 3).

### 3.2 Effects on lipid peroxidation

The levels of lipid peroxidation and MDA contents in the gastric of H/E‐treated control mice were significantly (*p* < 0.01) increased, compared to those in intact vehicle control mice. However, this increase in MDA content was significantly (*p* < 0.01) and dose‐dependently decreased in mice observed with all three doses of FBe. In addition, gastric lipid peroxidation induced by the HCl/EtOH mixture was also significantly (*p* < 0.01) inhibited after a single oral administration of RA 100 mg/kg, similar to that induced by FBe 200 mg/kg (Table 1).

### 3.3 Changes in CAT activity

The CAT activity in the gastric of mice receiving a H/E dose was significantly (*p* < 0.01) decreased, compared to the effects of mice with intact vehicle control. However, this decrease in CAT activity induced by the treatment of HCl/EtOH mixture was significantly (*p* < 0.01 or *p* < 0.05) and markedly inhibited after a single oral administration of RA 100 mg/kg and also after a single oral administration of FBe (300, 200, and 100 mg/kg), dose‐dependently. FBe 200 mg/kg showed similar inhibitory effects on the decrease in gastric CAT activity induced by the treatment of HCl/EtOH mixture compared to the effect of RA 100 mg/kg (Table 1).

### 3.4 Effects on SOD activity

The level of SOD activity in the gastric of H/E control mice was significantly (*p* < 0.01) decreased, while comparing to those in mice with intact vehicle control. However, this decrease in SOD activity was significantly (*p* < 0.01 or *p* < 0.05) and dose‐dependently inhibited in mice received different doses of FBe (300, 200, and 100 mg/kg). In addition, decreases in gastric SOD activities induced by the HCl/EtOH mixture were also significantly (*p* < 0.01) inhibited after a single oral administration of RA 100 mg/kg, similar to those induced by FBe 200 mg/kg (Table 1).

### 3.5 Effects on gastric nitrate/nitrite levels

The level of gastric nitrate/nitrite content of mice receiving a H/E dose was significantly (*p* < 0.01) decreased, when compared with intact vehicle control mice. However, this decrease in gastric nitrate/nitrite content was significantly (*p* < 0.01 or *p* < 0.05) normalized in mice after oral administration of FBe, dose‐dependently. In addition, RA at 100 mg/kg also favorably and significantly (*p* < 0.01) inhibited the effects of HCl/EtOH‐induced depletion in gastric nitrate/nitrite content compared to those in mice with H/E treatment and similar to that of FBe at 200 mg/kg (Table 1).

### 3.6 Changes on gastric mucosa histopathology

**Figure 4:**
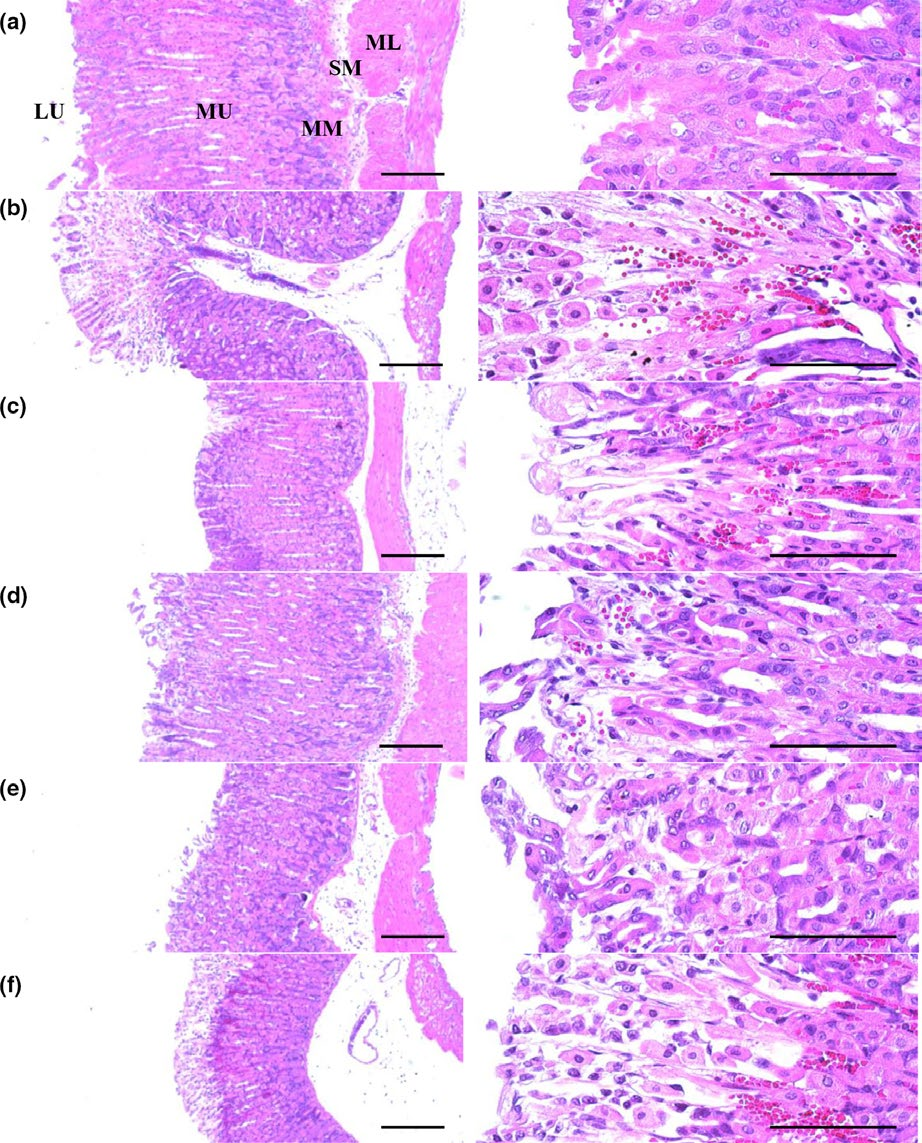
Representative histological images of the fundic mucosa, taken from intact or H/E‐treated mice. (a) Intact control (Distilled water 10 ml/kg and 5 ml/kg administrated mice). (b) H/E control (Distilled water 10 ml/kg and H/E 5 ml/kg‐treated vehicle control mice). (c) Reference (RA 100 mg/kg and H/E 5 ml/kg administrated reference drug‐treated mice). (d) FBe 300 (FBe 300 mg/kg and H/E 5 ml/kg treated the highest dosage experimental mice). (e) FBe 200 (FBe 200 mg/kg and H/E 5 ml/kg treated the highest dosage experimental mice). (f) FBe 100 (FBe 100 mg/kg and H/E 5 ml/kg treated the highest dosage experimental mice). FBe: Triple‐fermented barley extracts, test material; HCl: Hydrochloric acid; EtOH: Ethanol; H/E: HCl/EtOH (98% EtOH contains 150 mM HCl) mixture; RA: Ranitidine hydrochloride; MM: Muscularis mucosa; LU: Lumen; SM: Submucosa; ML: Muscular layer; MU: Mucosa layer. All hematoxylin and eosin stain. Scale bars = 180 μm

Ulcerative lesions were detected on the fundus as severe focal extensive superficial epithelial damage, desquamation of focal epithelium, congestion/hemorrhages, inflammatory cell infiltrations, and necrosis of gastric glands after treatment with the HCl/EtOH mixture. However, treatments with RA 100 mg/kg or FBe (100, 200, and 300 mg/kg) significantly inhibited these microscopic ulcerative lesions. Following histomorphometric and semiquantitative analysis, significant (*p* < 0.01) increases in invasion percentages of lesions and semiquantitative histological scores and decreases in periulcerative mucosa thicknesses were observed in mice with H/E control group compared to those of intact vehicle control mice; however, these markers were significantly (*p* < 0.01 or *p* < 0.05) normalized by the treatment of FBe (300, 200, and 100 mg/kg). In addition, significant (*p* < 0.01) decreases in invasion percentages of lesions and semiquantitative histological scores and increases in periulcerative mucosa thicknesses were observed in RA 100 mg/kg‐treated mice compared to those in H/E control mice. Upon treatment of FBe 200 mg/kg, mice showed similar inhibitory effects on the damage of histopathological gastric mucosa induced by the treatment of HCl/EtOH mixture, while comparing to those of RA 100 mg/kg‐treated groups (Table 2, Figure 4).

**Table 2:** Histomorphometrical analysis on the fundus, taken from intact or HE‐treated mice

Items (unit) groups	Histomorphometry (at sacrifice)		
	Invaded percentages of lesions (%)	Mean periulcerative mucosa thickness (μm)	Semiquantative scores (Max = 3)
Control			
Intact	1.29 ± 1.17	748.72 ± 126.79	0.50 ± 0.53
HE	71.43 ± 11.96^e^	304.81 ± 58.72^a^	2.63 ± 0.52^a^
Reference			
RA 100 mg/kg	28.39 ± 7.18^e^, ^f^	524.57 ± 82.22^a^, ^c^	1.50 ± 0.76^a^, ^c^
FBe treated as			
300 mg/kg	18.97 ± 5.90^e^, ^f^	604.91 ± 89.74^a^, ^c^	1.25 ± 0.46^b^, ^c^
200 mg/kg	29.86 ± 10.03^e^, ^f^	522.83 ± 51.24^a^, ^c^	1.63 ± 0.52^a^, ^c^
100 mg/kg	45.71 ± 10.70^e^, ^f^	411.43 ± 51.14^a^, ^d^	1.88 ± 0.64^a^, ^d^

Values are expressed mean ± *SD* of eight mouse samples.

Invasive Percentages of Lesions (%) = (Length of lesions on the crossly trimmed fundic walls/total thickness of crossly trimmed fundic walls) × 100.

Semiquantative scoring were divided into four degrees; 0 = normal intact mucosa, 1 = slight surface erosive damages, 2 = moderate mucosa damages and 3 = severe total mucosa damages.

FBe: Triple‐fermented barley extracts, test material; HCl: Hydrochloride; EtOH: Ethanol; HE: HCl/EtOH (98% EtOH contains 150 mM HCl) mixture; RA: Ranitidine hydrochloride.

^a^ *p* < 0.01 and ^b^ *p* < 0.05 as compared with intact control by LSD test.

^c^ *p* < 0.01 and ^d^ *p* < 0.05 as compared with HE control by LSD test.

^e^ *p* < 0.01 as compared with intact control by MW test.

^f^ *p* < 0.01 as compared with HE control by MW test.

## 4 DISCUSSION

Excess EtOH leads to erosion, ulcerative lesions, and petechial bleeding in the mucosa of the stomach and it is rapidly absorbed through gastric mucosa (Eastwood & Kirchner, 1974; Oyagi et al., 2010; Tarnawski et al., 1981). Excess ethanol also generates harmful ROS, superoxide anion, and hydroperoxy free radicals, involved in the pathogenesis of gastric mucosa (Dragland, Senoo, Wake, Holte, & Blomhoff, 2003; Huh et al., 2003; Oyagi et al., 2010). Gastric mucosa damage can be easily produced by the generation of toxic free radicals (Biswas et al., 2003; Naito et al., 1995) and disorders or decreases in gastric mucosa antioxidant defense systems have been involved in the pathogenesis and progression of gastric ulcers (Kwiecień et al., 2002). EtOH is often used to induce gastric ulcer in various model systems to study on the development of drug for human ulcerative disease (Szabo & Brown, 1987) and to evoke severe and rapid gastric damage together with administration of HCl (Alqasoumi et al., 2009; Huh et al., 2003; Lee et al., 2010; Oyagi et al., 2010). Various synthetic antiulcer drugs are currently available, and some of these including RA, are representative histamine H_2_ receptor antagonists specifically used to cure gastric ulcers. However, each of these drugs has simple‐to‐severe side effects (Miederer, 1986) including cardiac toxicity (Jakob et al., 2005), risk of pneumonia in hospitalized patients (Mallow et al., 2004), infectious diarrhea (Neal et al., 1994; Ruddell et al., 1980), the development of food allergies (Untersmayr et al., 2005), and thrombocytopenia (Bangia et al., 2011).

Various fermentation processes increase the bioavailability of phenolic compounds of barley and increase various antioxidant‐based pharmacological activities (Giriwono et al., 2010, 2011; Hokazono, Omori, Yamamoto, et al., 2010; Hokazono, Omori, & Ono, 2010; Iguchi et al., 2009; Kim et al., 2007). In our previous reports on the laxative effects of FBe on normal rats (Lim et al., 2018a) and loperamide‐induced constipated rats (Lim et al., 2018b), FBe showed favorable laxative effects at dose levels of 300, 200, and 100 mg/kg. In this study, three different doses of FBe (300, 200, and 100 mg/kg) were orally administered 1 hr before the HCl/EtOH mixture (5 ml/kg of 98% EtOH contains 150 mM HCl) treatment. One hour after treatment of HCl/EtOH mixture, the following changes such as the gross hemorrhagic lesion scores (mm^2^/gastric mucosa), fundic histopathology, gastric nitrate/nitrite contents, lipid peroxidation, and antioxidant defense systems (CAT and SOD activities) were observed, and the activity was compared with that of the synthetic antiulcer drug, a representative histamine H_2_ receptor agonist, RA 100 mg/kg (Grover et al., 2001) in this experiment. The doses of FBe, 100, 200, and 300 mg/kg, were selected, similar to those in our previous reports on the laxative effects of FBe on normal rats (Lim et al., 2018a) and loperamide‐induced constipated rats (Lim et al., 2018b), and the dose level of RA was also selected as 100 mg/kg according to a previous efficacy study (Grover et al., 2001).

The decrease or inhibition of gross hemorrhagic lesion areas is regarded as a valuable indication that test substances have favorable gastric mucosa protective effects based on previous efficacy studies conducted by other researchers (Mori, Hayashi, Iwashima, Matsunaga, & Saito, 2006; Süleyman et al., 2009). Reduced gross lesions indicate more favorable protective effects (Oyagi et al., 2010). The dose‐dependent decreases in gross lesion areas in mice after administration of FBe with three different doses, while comparing to those in mice with H/E control group suggest a favorable effects in protecting gastric ulcer. In particular, FBe also increased gastric nitrate/nitrite content and strengthened the antioxidant defense systems, decreased the level of gastric lipid peroxidation, but increased the activities of CAT and SOD. It indicates that oral administration of FBe (100, 200, and 300 mg/kg) showed favorable gastroprotective effects through the strengthening of the body's antioxidant defense system, at least under the conditions of this experiment. This study also demonstrated that FBe 200 mg/kg showed a similar gastroprotective effect to RA 100 mg/kg against HCl/EtOH‐induced gastric ulcer in mice, suggesting that FBe can be easily adjusted to patients suffering from various gastric damages at a dose level of 100 mg/kg, with respect to gastric damage in mice.

Ulcerative lesions have been detected histopathologically based on severe focal extensive superficial epithelial damage, desquamation of focal epithelium, focal hemorrhages/congestions, inflammatory cell infiltration, and necrosis of gastric glands and on the fundus after treatment with the HCl/EtOH mixture (Horiuchi, Wachi, & Seyama, 2010; Oyagi et al., 2010; Sánchez et al., 2006; Yesilada & Gurbuz, 2010). Changes in the histopathological images have been used as a valuable criteria index to confirm the gastroprotective effects of various candidates including medicinal herbs (Graziani et al., 2005; Oyagi et al., 2010; Yesilada & Gurbuz, 2010). In our results, HCl/EtOH‐associated microscopic ulcerative lesions were markedly inhibited by pretreatment with RA 100 mg/kg and by treatment with all three doses of FBe in a dose‐dependent manner. FBe 200 mg/kg showed similar histopathological effects to those of RA 100 mg/kg in this study, which corresponds well with other results in gross observation, gastric nitrate/nitrite levels, and antioxidant defense systems. These effective changes were also reconfirmed by histomorphometric analysis of the invasion percentages of lesions and periulcerative mucosa thicknesses and semiquantitative histological scores. Periulcerative mucosa thicknesses were significantly decreased and the invasion percentages of lesions and semiquantitative histological scores were markedly increased by treatment with HCl/EtOH, but they were favorably normalized by treatment with all three doses of FBe in a dose‐dependent manner and also after a single oral administration of RA 100 mg/kg similar to those with FBe 200 mg/kg.

## 5 CONCLUSION

By assessing the key parameters for protective effects against HCl/EtOH‐induced gastric damage in mice, the oral administration of 100, 200, and 300 mg/kg of FBe in this study, revealed favorable gastroprotective effects by strengthening the antioxidant defense systems. Our experimental results also showed that FBe 200 mg/kg had similar inhibitory effects against HCl/EtOH‐induced gastric damage in mice to those of RA 100 mg/kg, which is direct evidence that FBe can be easily adjusted to treat patients suffering from various gastric damages at a dose level of 100 mg/kg as for HCl/EtOH‐induced gastric damage in mice.

## CONFLICT OF INTEREST

The authors declare that there is no conflict of interest.

## ETHICAL APPROVAL

Ethical review: This study was approved by the Institutional Animal Care and Use Committee of Daegu Haany University (Gyeongsan, Korea) (Approval No. DHU2014‐086).

Informed consent: Written informed consent was obtained from all study participants.
